# Simulation of Far-Field Light Distribution of Micro-LED Based on Its Structural Parameters

**DOI:** 10.3390/ma15248854

**Published:** 2022-12-12

**Authors:** Wei Wei, Yiying Chen, Chenxi Wang, Xing Peng, Tang Tang, Zhizhong Chen

**Affiliations:** 1 School of Physics and Electronic Engineering, Yancheng Teachers University, Yancheng 224051, China; 2 State Key Laboratory of Artificial Microstructure and Mesoscopic Physics, School of Physics, Peking University, Beijing 100871, China

**Keywords:** micro-LED, far-field light distribution, luminous efficiency

## Abstract

To clarify how micro-LED far-field light distributions differ from Lambertian distributions owing to small-sized-structure effects, the light distribution of a micro-LED was simulated via the ray-tracing method in this study. Specifically, considering material absorption, far-field light distribution, and light-output efficiency, we studied micro-LEDs as a function of size. We found that the light distribution is the most uniform and the efficiency is the highest when the size is the smallest under certain conditions. Under other conditions, with increasing sapphire size, the luminous efficiency first increases and then decreases. The luminous efficiency is the highest when the thickness is 30 µm. Under certain other conditions, as the diameter of the micro-sphere structure on the sapphire increases, the luminous efficiency first increases and then decreases.

## 1. Introduction

Micro-LEDs (LEDs with sizes <100 µm [1]) are used in self-emitting displays, especially micro-projection displays. The contrast, efficiency, resolution, and response time of a micro-LED are typically high, and micro-LED displays outperform LCD and OLED displays in terms of brightness, resolution, contrast, energy consumption, service life, response speed, and thermal stability [2]. There is a surging demand for micro-LED display panels for smartwatches, phones, TVs, laptops, and augmented/virtual reality devices [3,4]. Nevertheless, such displays still face technical challenges, and some key technologies and process equipment have not yet been fully developed.

A traditional LED is a Lambertian light source with an uneven light intensity spatial distribution and a large light beam divergence angle [5]. The chip size of mini-LEDs ranges from 100 to 200 μm [3]. Mini-LEDs are thick; consequently, the Lambertian distribution leads to a small field of view, a lack of light uniformity, and other problems [6]. To overcome these problems, one solution is to increase the optical distance between the mini-LED backplane and the diffuser plate while keeping the array arrangement fixed [7]. Another is to compensate for the optical distance by using more mini-LEDs [8]. A third solution is local dimming with an integrated light-guiding plate [9].

LCDs and OLEDs perform comparably in terms of color gamut [10,11], resolution, response time [12], and power consumption. However, LCD displays have very limited viewing angles, which is a significant problem because they operate by blocking light and have intrinsic viewing obstacles at certain angles. When the angle is even slightly excessive, it is impossible to see the original color (or sometimes anything at all). Solutions to this problem include novel polarization converters based on reflective metal gratings and polarized beam separators [13] and the use of a unique roll-to-roll large-scale high-transmission wide-angle diffuser film [14]. An LCD also has a backlight layer; therefore, light can easily leak from the gap between the screen and the border [15].

A micro-LED exhibits none of these disadvantages. It has fields of view on both the side and front, although the side field of view is limited to a particular angle. There are ways of improving the light extraction efficiency of small-angle micro-LEDs [16,17,18], but when a micro-LED is viewed from a low angle on the side, an optimal view may not be possible. Researchers have presented calculations without considering the absorption exhibited by the micro-LED material. However, those calculations may deviate from experimental data because they do not consider the absorption. In this study, we use the uniform light distribution of micro-LEDs to increase the field of view by performing calculations considering the material absorption.

## 2. Design Concept

The far-field light distribution of most modern LED chips is a Lambertian distribution. This distribution causes approximately 80% of the light to fall within 120° because the cell size is so large and most light is totally reflected by the top and bottom sides. The optical intensity *I* is highest when the angle *θ* measured from the normal is close to 0° and decreases as *θ* approaches 90° [19,20]:

*I* = *I*_0_ cos *θ* (−π/2 ≤ *θ* ≤ π/2)

where *I*_0_ is the maximum intensity.

Existing LED-chip (or lamp-bead) composite Lambertian distributions cannot meet the requirements of display mixing: intense light, a small middle-edge light intensity, and an effective intensity that is concentrated within a 120° angle. To utilize the three primary colors, mixed light requires a certain distance; an insufficient distance affects the user experience, including in the case of TV screens. The current solution is to use a mixed lens; however, the lens itself also occupies space, so the thickness reduction is limited. The light distribution from an LED chip (or bead) in a display screen should be highly uniform to make it easier to realize an ultra-thin screen and thereby improve the user experience.

To date, there are only two ways to paint black light-absorbing material on the display screen: on the bottom surface between pixels to reduce the reflection of external incident light from the bottom surface and improve the contrast, or on the sides of the pixels themselves. For a modern display with a large proportion of chips or beads, the sides of each pixel must be coated to absorb light from a small angle to prevent crosstalk between adjacent pixels. These methods reduce the LED’s luminous efficiency. As the size and height of LED chips or micro-beads decrease, larger light angles and more uniform light distributions are obtained. To obtain a better light angle and a highly uniform light distribution, it is necessary to simulate the optical properties of micro-LEDs.

Micro-LED chips produce a more even distribution; developing highly homogeneous micro-LED optical-design technology is therefore an urgent scientific and technological challenge. Direct measurements have shown that the secondary light distribution curve of micro-LEDs is non-Lambertian [1,2,5]. Bayneva [21] simulated the light distribution using Tracepro. They applied the ray-tracing method in the early stages and found that as the size of the micro-LED decreased, the micro-LED far-field light distribution stopped following a Lambertian distribution [1,22].

Changing the chip structure can affect the light distribution of the chip; simulation results showed that when the size was reduced to 10 μm, the far-field optical distribution differed significantly from a Lambertian distribution, and its far-field distribution was more uniform. An experimental study by Xu et al. [23] further supported the simulation results [24].

The aforementioned studies have shown that the far-field light distribution of the vertical structure differs from a Lambertian distribution, indicating that the light distribution of the LED can be improved through the design of an appropriate chip structure.

Furthermore, the existing research focuses on the influence of different micro-LED chip sizes on the far-field light distribution and output efficiency of micro-LEDs. At present, the absorption of materials and the influence of microstructure on the far-field light distribution and light output efficiency of LEDs are rarely considered.

Keeping the layer thickness of different materials constant, the chip size of the micro-LED cell was changed from 10 µm to 20, 30, 40, and 50 µm; the refractive and absorption indices of the different materials are listed in Table 1. The geometric optics simulation calculation adopted in this study does not consider diffraction and interference, which cause great light-field redistribution due to size change. Therefore, regardless of the material used, the refractive and absorption indices of the materials did not change according to micro-LED size. The refractive index of (Al_2_O_3_)_2_ (1.7) was the lowest among all materials; that of the active layer (2.54) was the highest. ITO had an absorption index of 0 mm^−1^; the highest absorption index (25 mm^−1^) was that of the active layer [24,25,26]. The wavelength simulated and calculated in this paper is 450 nm.

**Figure 1:**
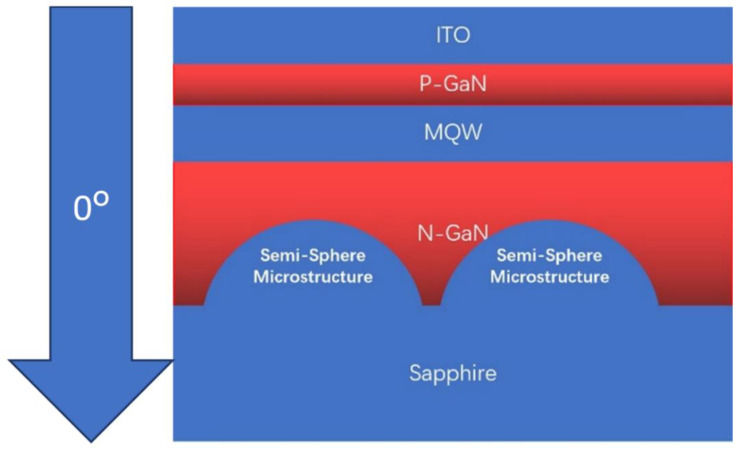
The structure of a micro-LED cell and the direction of 0°.

In this study, based on the ray-tracing method, the Monte Carlo method was used to simulate the calculation, and Tracepro 7 software was used to simulate the calculation. The calculation principle is based on geometric optics, specifically considering the reflection and refraction laws of light at the interface. Wave optics, diffraction, and interference are not considered, so the diffraction effect is not considered. The active layer is assumed to be a quantum well layer, and it is assumed that the light emitted from the surface of the well layer at this time is uniformly distributed. According to these assumptions, the ordinary LED distribution we obtained is a Lambert distribution, which is in good agreement with the experimental data. This study employed a 3D simulation. The 0°-direction of the far-field light distribution provided the vertical direction, and the 0° direction in Figure 1 was increased. Keeping the layer thickness of different materials constant, the semi-sphere micro-structure size of the Al_2_O_3_ film was changed from 1 µm to 2, 3, and 4 µm, respectively, and the refractive and absorption indices of the different materials are listed in Table 2.

Keeping the layer thickness of different materials constant, the Al_2_O_3_ film size was changed from 10 µm to 30 and 50 µm, respectively. The refractive and absorption indices of the different materials are listed in Table 3. The device structure size was designed according to the sizes mentioned in Table 1, Table 2 and Table 3.

## 3. Results and Analysis

**Figure 2:**
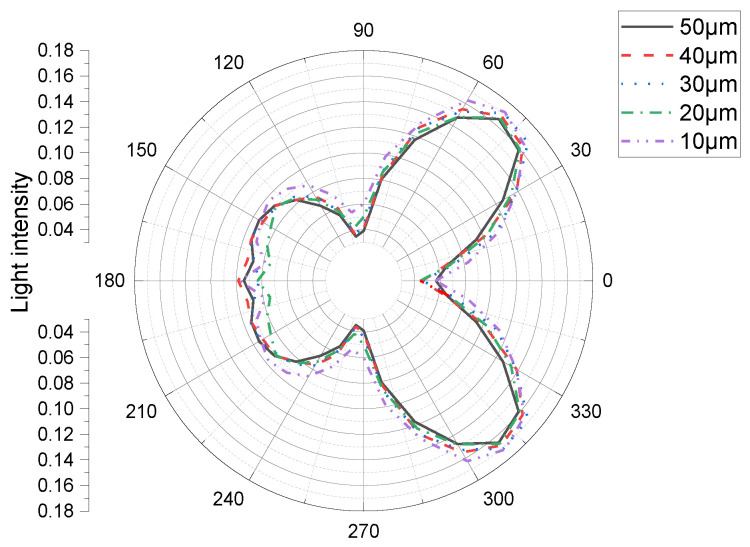
Simulated light distribution of a constant-thickness micro-LED cell for various chip sizes.

The relationship between the light distribution and light-output efficiency of a micro-LED cell with a constant Al_2_O_3_ film thickness when changing the size of the micro-LED cell is shown in Figure 2, where the light efficiency is represented by the area enclosed by the curve. In the simulation, we changed the chip size from 10 µm to 20, 30, 40, and 50 µm. The overall pattern exhibited four lobes. The luminous efficiency and light distribution were approximately the same at 0° and 180°. When the micro-LED cell size was smaller (when the cell size was 30 µm, which is larger than 20 µm, and the semi-sphere micro-structure was different), the total enclosed area (and thus the light efficiency) was larger (Table 4).

The maximum light intensity was 0.653 at a size of 10 µm. On any single side of the micro-LED cell, the reflective intensity was highest at the center. As can be observed from the figure, the far-field light distribution of the micro-LED shows that the light output in the middle is small, and the light output on both sides is large. This is because as the size of the micro-LED decreases, the area of the front decreases, the light output of the front decreases, and the ratio of the area of the side to the area of the front increases. Most of the light comes out of the side; thus, the light on the side increases. Similar results were obtained by Korean researchers; however, they did not consider the material absorption [5].

**Figure 3:**
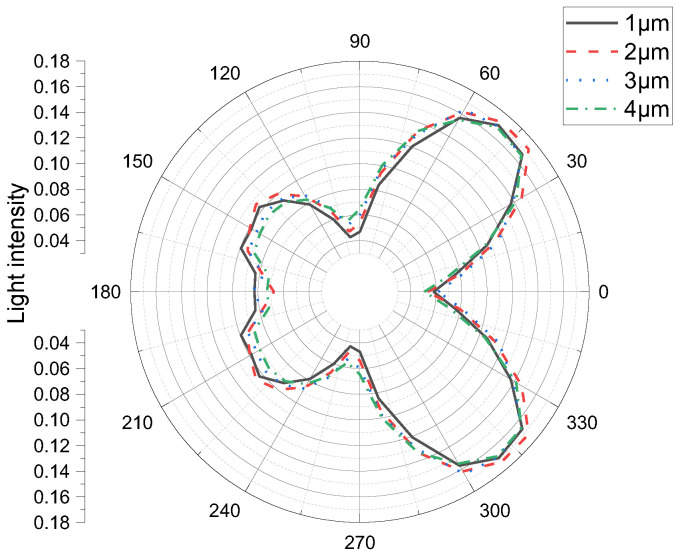
Simulated light distribution of a constant-thickness micro-LED cell for various semi-sphere micro-structure sizes.

The relationship between the light distribution and light-output efficiency of a micro-LED cell with the same size as the semi-sphere micro-structure on the Al_2_O_3_ film was also examined. In the simulation (Figure 3), we changed the diameter of the semi-sphere micro-structure on the Al_2_O_3_ film from 1 µm to 2, 3, and 4 µm. When the diameter was 1 µm, among the light efficiencies of the 10-, 30-, and 50-µm Al_2_O_3_ films, that of the 30-µm film was the largest (Table 5, Figure 3). On any single side of the micro-LED cell, the reflective intensity was highest at the center.

**Figure 4:**
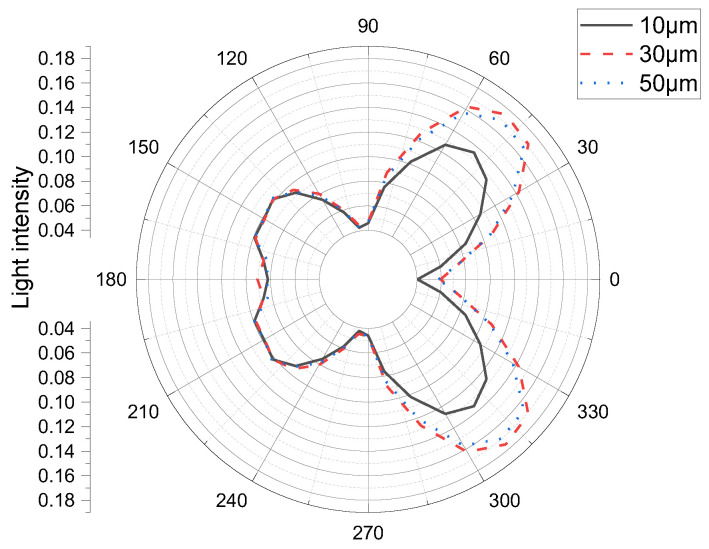
Simulated light distribution of a constant-size micro-LED cell for various (Al_2_O_3_)_2_ film thicknesses with a 10-μm chip size.

**Figure 5:**
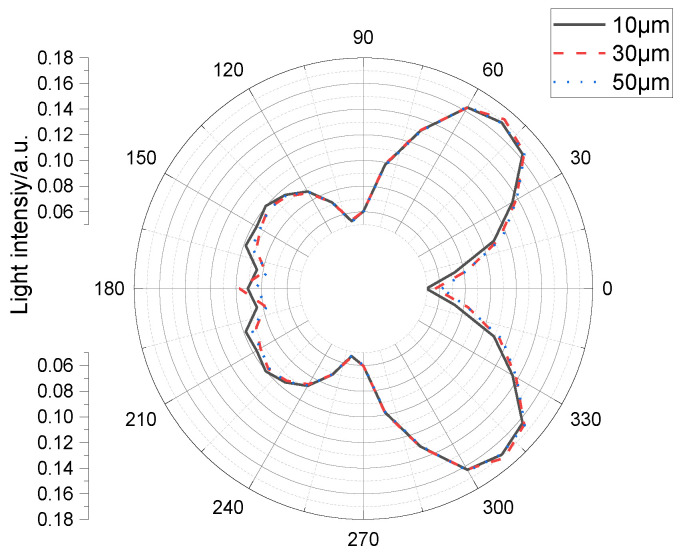
Simulated light distribution of a constant-size micro-LED cell for various (Al_2_O_3_)_2_ film thicknesses with a 30-μm chip size.

The real efficiencies of the 10-, 30-, and 50-µm Al_2_O_3_ films were 0.6444, 0.64429, and 0.644438, respectively, as listed in the bottom right corner of Table 6. It is worth noting that, because of the relationship between significant figures, the first three decimal digits are the same (as listed in Table 6), despite the actual values being slightly different. Figure 4 and Figure 5 show that the luminous efficiency of the micro-LED increases with the sapphire layer thickness. This is because with increasing sapphire size, the angle from the light emitted from the micro-LED active layer to the front light-emitting surface becomes progressively smaller, which prevents the front light from being reflected back by the total reflection. However, as the size continues to increase, the material absorbs more light, and the efficiency of the front light emittance decreases. Further, with increasing sapphire size, the light from the active layer of the micro-LED shines increasingly more on the side, resulting in more side light. Thus, the sidelight efficiency also increases. However, with a further increase in size, the material absorbs more light, and the sidelight efficiency is reduced. Finally, it is concluded that the luminous efficiency of the micro-LED is highest when the thickness of the sapphire is 30 µm (Table 6, Figure 4 and Figure 5).

The possible reasons for these results are as follows: When the chip thickness is small, the structure change plays a major role in the light output efficiency. At this time, the light output efficiency is generally high. As the thickness increases, the material’s light absorption increases, which leads to a decline in efficiency. When the thickness is 30 µm, the two effects cancel each other out, so the light output efficiency of the micro-LED chip is at its maximum.

## 4. Conclusions

In this study, considering material absorption, far-field light distribution, and light-output efficiency, we investigated micro-LEDs as a function of size. We found that the light distribution is mostly uniform, and the efficiency is highest when the size is smallest. Under other conditions, with increasing sapphire size, the luminous efficiency first increases and then decreases. When the thickness is 30 µm, the luminous efficiency is the highest (0.656). Under other conditions, as the diameter of the micro-sphere structure on the sapphire increases, the luminous efficiency first increases and then decreases. In addition, the far-field light distribution of the micro-LED does not follow a Lambertian distribution (the Lambertian distribution light beam is 120°), and its light beam angle is approximately 150°, which can meet the requirements of future displays.

In the future, micro-LED chips with appropriate chip sizes and structures will be able to provide the intense, uniform non-Lambertian light distribution required for display mixing. On the one hand, an ultra-thin screen can be achieved; on the other hand, the field of view can be increased. Using the data and findings of this study, more efficient LEDs could be fabricated and achieve better luminous effects, improving their applicability in the automotive, medical, and fiber-optic fields, among other fields.

Combined with the design idea presented in this paper, considering light absorption, a far-field light distribution and light-output efficiency that meet the display requirements can be achieved by optimizing the appropriate structural parameters of micro-LED devices, i.e., by improving the light energy utilization efficiency of micro-LEDs, realizing the wide-angle light emission of micro-LED displays, and upgrading the carbon neutralization technology of micro-LED displays.

## Figures and Tables

**Table 1:** Refractive and absorption indices of materials in micro-LEDs in the 10–50-μm chip-size range [5,24,25,26,27,28].

Material	Thickness	Refractive Index	Absorption Index [mm^−1^]
Semi-sphere micro-structure	Φ 3 µm	1.70	0.004
Sapphire	5 µm	1.70	0.004
ITO	300 nm	1.50	0
p-GaN	150 nm	2.45	2.300
Active layer (MQW)	100 nm	2.54	25
n-GaN	6.75 µm	2.45	2.3

**Table 2:** Refractive and absorption indices of materials in a micro-LED unit with a 10-μm chip and a 1–4 μm semi-sphere micro-structure range [5,24,25,26,27,28].

Material	Thickness (µm)	Refractive Index	Absorption Index [mm^−1^]
Semi-sphere micro-structure	Φ1–4	1.70	0.004

**Table 3:** Refractive and absorption indices of materials in a micro-LED unit with a 10-μm chip, 3-μm semi-sphere micro-structure, and 10–50-μm Al_2_O_3_ range [5,24,25,26,27,28].

Material	Thickness	Refractive Index	Absorption Index [mm^−1^]
Semi-sphere micro-structure	Φ3 µm	1.70	0.004
Al_2_O_3_	10–50 µm	1.70	0.004
ITO	300 nm	1.50	0
p-GaN	150 nm	2.45	2.300
Active layer	100 nm	2.54	25
n-GaN	6.75 µm	2.45	2.300

**Table 4:** Light efficiency of a constant-thickness micro-LED cell for various chip sizes with a 30-μm Al_2_O_3_ film and a 3-μm semi-sphere micro-structure size.

Chip Size [μm]	Light Efficiency
10	0.653
20	0.590
30	0.605
40	0.597
50	0.572

**Table 5:** Light efficiency of a constant-size micro-LED cell for various semi-diameters of the semi-sphere micro-structure at various (Al_2_O_3_)_2_ film thicknesses.

Semi-Diameter of the Semi-Sphere Micro-Structure at a 10-μm-Thick (Al_2_O_3_)_2_ Film	Light Efficiency	Semi-Diameter of the Semi-Sphere Micro-Structure at a 30-μm-Thick (Al_2_O_3_)_2_ Film	Light Efficiency
0.5	0.543	0.5	0.633
1	0.518	1	0.656
1.5	0.653	1.5	0.653
2	0.644	2	0.644

**Table 6:** Light efficiency of a constant-size micro-LED cell for various (Al_2_O_3_)_2_ film thicknesses at various semi-diameters with a 10-μm chip size.

(Al_2_O_3_)_2_ Film Thickness at a 0.5-μm Semi-Diameter of the Semi-Sphere Micro-Structure	Light Efficiency	(Al_2_O_3_)_2_ Film Thickness at a 1-μm Semi-Diameter of the Semi-Sphere Micro-Structure	Light Efficiency	(Al_2_O_3_)_2_ Film Thickness at a 1.5-μm Semi-Diameter of the Semi-Sphere Micro-Structure	Light Efficiency	(Al_2_O_3_)_2_ Film Thickness at a 2-μm Semi-Diameter of the Semi-Sphere Micro-Structure	Light Efficiency
10	0.543	10	0.518	10	0.653	10	0.644
30	0.633	30	0.656	30	0.653	30	0.644
50	0.617	50	0.655	50	0.652	50	0.644

## Data Availability

An experimental study by Xu et al. [23] further supports the simulation results [24]. Link to: https://doi.org/10.1109/JPHOT.2019.2962184 and https://link.springer.com/article/10.1134/S1063782608110195. Most of the simulation conclusions in this paper can be confirmed by the data of other researchers that have been published. The specific document name is: “Chip size-dependent light extraction efficiency for blue micro-LEDs”. The link is: https://koreascience.kr/article/JAKO201909862999978.page.
